# Vocalisations are coupled with movement of all limbs throughout infancy

**DOI:** 10.1038/s41598-025-28388-6

**Published:** 2025-12-29

**Authors:** Joanna Duda-Goławska, Zuzanna Laudańska, Karolina Babis, Anna Malinowska-Korczak, Alicja Radkowska-Palińska, Przemysław Tomalski

**Affiliations:** 1 Neurocognitive Development Lab, Institute of Psychology, Polish Academy of Sciences, Warsaw, Poland; 2 Department of Child and Adolescent Psychiatry, Heidelberg University Hospital, Heidelberg University, Heidelberg, Germany

**Keywords:** Infancy, Vocalisations, Limb movements, Speech-gesture system, Motor-vocal coupling, Developmental biology, Evolution, Neuroscience, Physiology

## Abstract

Early speech development involves learning complex motor actions under constantly changing body proportions and biomechanics. However, the motor underpinnings of infant vocal production are largely unknown. The key question is whether vocal production (a complex motor action) is related to limb movements at the early stages of learning to speak. We tracked the development of coupling between limb movements and vocalisations in infants at 4, 6, 9 and 12 months of age. Limb movements were tightly coupled with vocalisation onsets at all time points across infancy. The initiation of speech-like vocalisations in early life is accompanied by movements of legs and arms. These findings suggest that the coupling of limb movements with vocalisation could be a potential mechanism supporting vocal production in early infancy, as well as a likely precursor to the emergence of gestures.

**Supplementary Information:**

The online version contains supplementary material available at 10.1038/s41598-025-28388-6.

## Introduction

Language development in infancy is marked by the rapid acquisition of speech sounds and words. While research has predominantly focused on perception, comprehension, and vocabulary growth^1^, prelinguistic vocal development is fundamentally characterised by continuous vocal production, enabling infants to gradually build a repertoire of articulated sounds and practice the complex motor actions related to production^2^. The maturation of the vocal apparatus—including significant changes in the musculoskeletal and neural structures of the vocal tract—is a necessary precursor to spoken language acquisition^3^. Thus, understanding prelinguistic vocal development requires close attention to the organisation of motor production^3^. However, the motor foundations of vocal production have not been systematically examined as a developmental basis for speech^1,4^.

Beyond the vocal apparatus itself, vocal production depends on broader bodily coordination, involving biomechanical links among vocal, respiratory, and motor systems^5^. In adults, limb movements of the upper limbs can influence vocalisation by modulating rib cage dynamics and altering respiratory flow^5–9^. These effects are likely mediated by muscle groups that simultaneously engage the arms and insert into the rib cage^10–13^. Emerging evidence also suggests that this coupling extends to leg movements^14,15^, but the potential involvement of limbs in prelinguistic vocal production remains understudied.

In infancy, rhythmic manual activity frequently co-occurs with vocalisations, forming what has been described as vocal-motor babbling^16–22^. This phenomenon points to a potential developmental coupling between motor and vocal systems. Yet, the origins and scope of this coupling are not fully understood. Iverson and Fagan^20^ identified a developmental trend between 6 and 9 months of age, with increasing coordination between arm movements and vocalisations alongside a decline in leg, torso or head movements during vocal production. However higher coordination was specifically linked to the babbling stage of vocal development. Furthermore, Ejiri and Masataka^19^, in a longitudinal case study (N = 4), found that vocalizations frequently co-occurred with rhythmic actions, particularly during the month preceding the onset of canonical babbling (i.e., the production of well-formed syllables consisting of a consonant and a vowel). More recently, Borjon et al.^23^ reported concurrent arm and head movements with vocalisations in infants aged 9 to 24 months (leg activity was not measured). Notably, arm movement velocity was temporally aligned with the onset of vocalisations across all age points, with this alignment becoming more precise with age. Pouw et al. hypothesised that such vocal-motor babbling – arm movements that are temporally synchronised with vocalisations – could be understood as vocal-entangled gestures and potentially result from the biomechanical interactions between muscles involved in arm movement, respiration, and vocal production^6–9^. In our study, we refer to this temporally aligned activity across limbs and vocal output as coupling, in a behavioural sense, to describe structured temporal overlap in motor and vocal activity observable in accelerometer and acoustic data.

The early motor-vocal coupling may also reflect broader mechanisms of sensorimotor integration and timing. Iverson^1^ has proposed that the development of vocal control is deeply intertwined with motor development. Furthermore, theoretical frameworks such as the Frame-then-Content model^24^suggest that rhythmic jaw movements provide a motor scaffold for early vocalisations, aligning with findings of repetitive limb movements during babbling. Rhythmic motor acts—whether limb, head, or jaw—may offer infants internally generated patterns that also support vocal exploration (e.g^25^.). These behaviours may also serve as early precursors to gesture-speech coupling seen in later development^26^, pointing to a shared developmental system for multimodal communication. Given the limited differentiation of motor systems (and especially different effectors) in early infancy, it is plausible that both upper and lower limbs participate in this exploratory coupling.

Moreover, postural control may enable more stable conditions for the emergence of coordinated motor–vocal behaviour^27,28^. Body positioning, especially the shape of the vocal tract, can affect both vocal production^29,30^ and limb movements^31^, leading to developmental changes in motor–vocal coordination when infants reach new milestones in their gross motor development. For example, upper limbs are involved in the forearm support position (prone), which is a frequent body posture around 6 months of age^32^. This dramatically changes when infants reach an independent sitting position (which usually happens between 6 and 9 months of age)^33^, which is the first upright body posture that frees arms from their role in postural support but constrains legs in a relatively fixed position. Whether such motor–vocal coupling emerges earlier in development, prior to the establishment of postural control necessary for upright vocal tract positioning, remains unknown. Moreover, it is unclear whether coupling is specific to the upper limbs or whether the lower limbs are also involved.

For these reasons in this study we investigated whether motor-vocal coupling is present in infants as young as 4 months of age, whether it involves both arm and leg movements, and how this coupling evolves across the first year of life. We analysed the acceleration of limb movement around the onset of a vocalisation in a longitudinal design at four developmental time points, from 4 to 12 months of age. In order to capture spontaneous patterns of behaviours, we used a semi-naturalistic design – free-flowing infant-parent interactions during a common daily activity of book-sharing. Infants were not constrained in terms of posture or limb use, which enabled the emergence of spontaneous, self-initiated motor and vocal behaviours. By “spontaneous vocal production,” we refer to infant vocalisations produced freely in this naturalistic context, without direct elicitation, instruction, or experimental prompting. Book-sharing activity elicits frequent vocalisations already in infancy (see ^34^, but it is not characterised by clearly predefined limb movement patterns (see ^35^) such as rhythmic arm movements during rattle-shaking^36^, which could significantly alter the baseline level of limb movements. Our measurement time points reflect significant changes in infant gross motor development and most frequent body positioning. As measured in the same cohort by Duda-Goławska et al.^37^, at 4 months infants spent most of the recording time in supine position, at 6 months in prone position and at 9 and 12 months in sitting.

## Materials and methods

### Participants

Participants included 104 White infant-parent dyads (41 girls) who were invited to the lab when infants were 4 (T1), 6 (T2), 9 (T3) and 12 (T4) months old (see Table 1). 83 dyads participated in a minimum of three visits, and out of them, 48 dyads contributed data at all 4 time points (missed visits are mostly due to COVID-19-related restrictions as data collection was conducted between 2020 and 2023). All infants were of Polish origin. The sample was recruited from urban and suburban environments in Warsaw, Poland (> 1.8 million inhabitants) and the surrounding metropolitan area (> 3 million inhabitants), which is characterised by limited ethnic diversity. Participants were from predominantly middle-class families, as indicated by caregiver self-reports of household income and education, aligned with OECD criteria for middle income (75–200% of the national median). In Poland during the study period (2020–2023), this corresponded to monthly net household incomes of approximately 5,000–12,000 + PLN. In our sample, most families reported incomes within this range, with the largest proportion (≈40%) in the 8,000–11,999 PLN bands and 32% reporting 12,000 PLN or higher. Only a small minority (≈5%) reported below 5,000 PLN. The majority (90%) of the caregivers had completed higher education: 3 held a PhD degree, 81 held a master’s degree, 10 held a bachelor’s, and 4 completed high school (6 missing data).

**Table 1:** Infant visits and vocalisations by age.

Time Point	Age (M ± SD	Age range	N Infants	N Vocalisations	N Vocalisations Per Infant (M ± SD)	Vocalisation Duration (ms, M ± SD)	Recording Duration (s, M ± SD)
T1: 4 mo	4.36 ± 0.29	3.9–5.2	61	2095	34 ± 33	593 ± 483	313 ± 29
T2: 6 mo	6.59 ± 0.40	6.0–7.8	75	2423	32 ± 24	589 ± 537	313 ± 30
T3: 9 mo	9.06 ± 0.35	8.2–10.0	69	1764	26 ± 19	547 ± 460	315 ± 40
T4: 12 mo	12.14 ± 0.52	11.0–14.5	72	2247	31 ± 19	638 ± 577	325 ± 78

Number of visits and vocalisations from infants participating at each age point. An individual infant could contribute data only once at each specific age.

All included infants were reported by caregivers to be typically developing: they were born at or near term (≥ 37 weeks of gestation; one infant born at 35 weeks with no subsequent complications was retained), had no diagnosed neurological, developmental, or sensory disorders, and no major perinatal risk factors such as very low birth weight, significant birth complications, or extended NICU stay were reported. Apgar scores were predominantly between 9–10 at 1 min (one infant with Apgar 5 but no sequelae or NICU stay was retained). All caregivers also confirmed that they had no concerns about their child’s development at the time of participation.

Overall, in 102 cases, the same caregiver interacted with the infant during all visits (101 mothers, 1 father). In two cases, because of the availability to schedule an appointment, different parents interacted with the child at different times. In some cases, two caregivers (both parents or a parent and a non-parental caregiver) were present during the visit, but only one of them was in the testing room interacting with the child during recording. The other caregiver stayed in the adjacent room and interacted with the child only during breaks in the recording (e.g., during feeding). All caregivers gave written informed consent before testing. For their participation, infants received a diploma and a small gift. The study conformed to the Declaration of Helsinki and received clearance from the Research Ethics Committee at the Institute of Psychology of the Polish Academy of Sciences (decision no. 10IV/2020).

We have excluded 41 out of 318 participants’ visits due to an infant’s refusal to wear motion trackers or technical problems with motion tracking, microphone, or synchronisation. The mean ages at each time point were: 4 months (*M* = 4.36, *SE* = 0.28), 6 months (*M* = 6.61, *SE* = 0.39), 9 months (*M* = 9.12, *SE* = 0.41), and 12 months (*M* = 12.17, *SE* = 0.51).

### Experimental design

We conducted a longitudinal study of the coupling between vocalisations and limb movement, testing in laboratory settings the same infant-caregiver pairs at four visits (T1-T4, around the age of 4, 6, 9, and 12 months). We measured spontaneous vocal and motor activity during semi-structured infant-caregiver social interactions.

The recording of interactions took place within a laboratory room, on a carpeted play area. Upon the family’s arrival, an experimenter explained the study protocol and obtained written informed consent from the parent (legal guardian). Once the infant was familiarised with the laboratory, the wearable motion trackers and head cameras were fitted on the infant and caregiver (data not reported here). Following this, a series of parent–child interactions with different sets of age-appropriate toys took place. The sets for infants aged 4 and 6 months differed slightly from those designed for infants aged 9 and 12 months, aiming to sustain their engagement. There were 6–7 tasks during each meeting (randomised among participants and across testing sessions), but here we only focus on the book-sharing task that lasted approx. 5 min. In the book-sharing task, infants and parents were provided with a set of infant books. At T1 and T2, there were three small picture books: one with nursery rhymes, one with big pictures of animals and people and one with pictures and onomatopoeic words. At T3 & T4, infants and parents were given one bigger book with pictures and onomatopoeic words and one smaller book with animal pictures, nursery rhymes about animals and tactile elements. The dyadic body positioning during the task was not predefined. Both the infant and the caregiver were free to move around the room and change positions as they preferred. The book-sharing task was naturalistic and unstructured: both partners were free to interact with the books in any manner they chose. As a result, the type and intensity of movements varied across participants, which helped to capture the typical range and intensity of movements during naturalistic infant-parent play. Caregivers were simply instructed to play with their infants as they normally would.

### Equipment

**Fig. 1:**
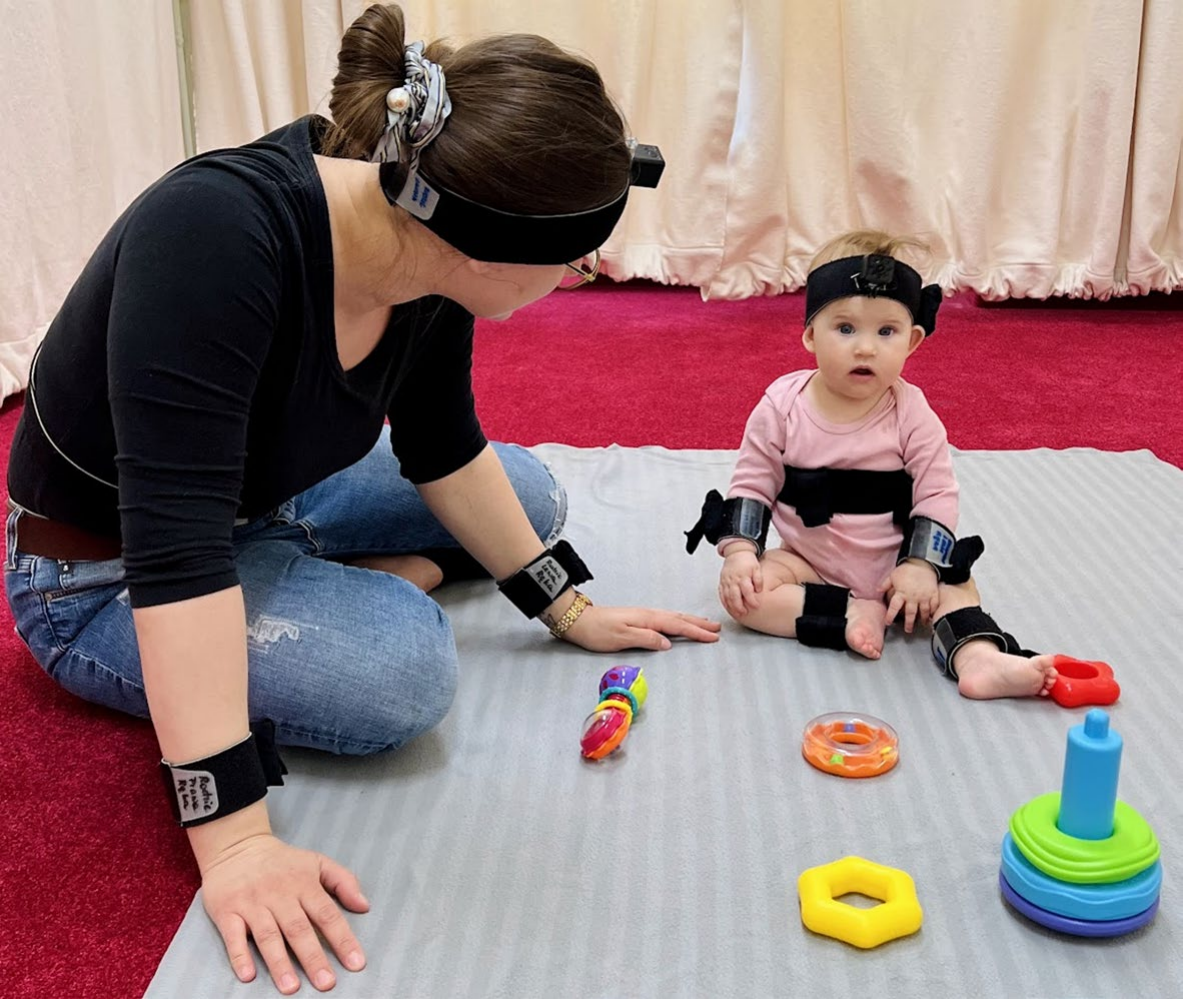
Sensor placement on the infant and caregiver. The photo shows the placement of all 12 sensors used in the study. Image provided by the Babylab, Institute of Psychology, Polish Academy of Sciences, with written consent from the parent (legal guardian) for publication.

Three remote-controlled CCTV colour cameras in HD quality recorded the interactions. The audio signal was captured at 44,100 Hz using a high-grade cardioid condenser microphone (Sennheiser e914), positioned beneath one of the cameras and synchronised with the video system. Body movements of the infant and the caregiver were recorded at 60 Hz using wearable motion trackers (MTw Awinda, Xsens Technologies B.V.), connected wirelessly via an Awinda station receiver and managed in real time with the MT Manager Software (Xsens Technologies B.V.), which ensured precise synchronisation of the sensors. All motion data were collected using a single computer. In total, 12 sensors were used: placed on the infant’s arms, legs, head, and torso, and on the caregiver’s arms, head, and torso. For the purposes of this study, we report only the data from two pairs of sensors placed on the infant’s arms and legs, as shown in Fig. 1 (image provided by the Babylab, Institute of Psychology PAS, with written consent from the caregiver or legal guardian for publication). Sensors were attached symmetrically on both limbs using soft elastic bands positioned just above the wrists and ankles to enable direct comparison between limbs.

**Fig. 2:**
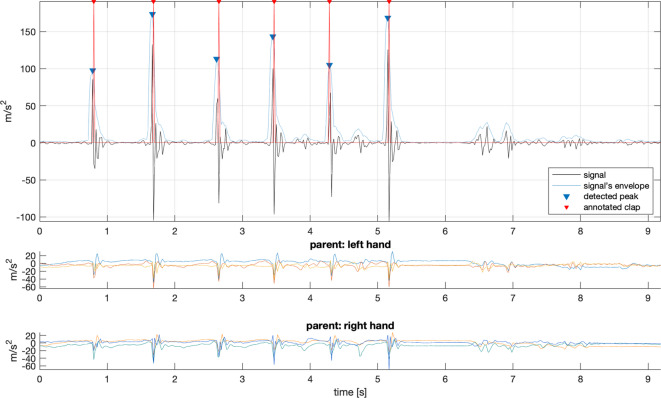
Illustration of synchronisation between IMU sensor signals and annotation. The figure presents a single synchronisation event consisting of five claps. The top plot shows the average acceleration of the caregiver’s left and right hands. The bottom plots display the individual 3-axis accelerometer signals from the left and right hand sensors.

Sensor and audio data were additionally aligned using hand claps performed by the caregiver at the beginning of each session. The acoustic peaks generated by the claps were clearly identifiable in both the audio recordings and the sensor signals. Synchronisation was achieved by calculating the delay between the audio and the averaged accelerometer signals from the caregiver’s hands—see Fig. 2**.**

Sensor signal segments exhibiting abnormally high amplitude values—identified using thresholds determined from events when the child threw the sensors—were considered artefacts and excluded from further analysis. These segments were brief (up to 0.6 s) and accounted for only a negligible portion of the data.

### Coding of infant vocalisations

For each interaction session, infants’ vocalisations were coded off-line using PRAAT software^38^. Annotations were saved to TextGrid format files. Two coders marked the onsets and offsets of each vocalisation at the utterance level. An utterance was defined as a vocalisation occurring on one expiration cycle^39,40^. Coding relied primarily on auditory–perceptual judgement, aided by visual inspection of the waveform and spectrogram when needed, consistent with established protocols in infant vocal development research^41^. All prelinguistic vocalisations were coded: protophones (speech-like, non-cry/non-laugh/non-vegetative infant vocalizations such as squeals, vowel-like sounds, growls, whispers, yells, grunts), syllables, and (proto-)words (based on^42^). “Syllables” included both canonical and marginal syllables. Vowel-only productions without consonantal elements were classified as protophones. Breathing sounds were not considered vocalisations. To calculate inter-rater agreement, 86 recordings were double-coded, and *Cohen’s kappa* was 0.85. Laughing and crying were not analysed for the aims of the present study. Good quality of recorded sound allowed for a clear identification of vocalisation onset even in the case of overlap with caregiver’s speech. For a full overview and detailed descriptive data on infant vocal behaviors see^34^. The example of audio signal, synchronised with the corresponding accelerometer (ACC) signal, is shown in Fig. 2.

### Data pre-processing

IMU (Inertial Measurement Unit) data from sensors placed on both wrists and ankles of an infant were processed in MATLAB^43^ and EEGlab^44^ using in-house scripts. Further analysis focused on the selected acceleration signals. The IMU tracking system, which measures the user’s orientation, operates wirelessly through WiFi; however, occasional issues with WiFi connectivity led to missing values in the IMU data. Internal features of the IMU sensors and automatic adjustments in the sampling rate from 60 to 40 Hz primarily caused these missing values. To ensure the comparability of time series data, missing values in the packages were interpolated using the Matlab function *interp1* with the ‘spline’ parameter. Upon detecting a lower sampling rate, the signal underwent resampling using the resample Matlab function. In addition, we calculated the magnitude of the three-dimensional acceleration vector at each time point^45^ using the formula (Eq. 1).

1 $$Acc(t) = \sqrt{{x(t)}^{2} +{y(t)}^{2}{ + z(t)}^{2}},$$

where $x,y,z,\in {\mathbb{R}}^{1x{\mathbb{N}}}$, and the variables $x(t),y(t),z(t),$ represent the accelerations along the three spatial dimensions over time.

Next, the signals underwent filtering using a 2nd order, 1 Hz cut-off highpass Butterworth filter.

### Movement data pre-processing

**Fig. 3:**
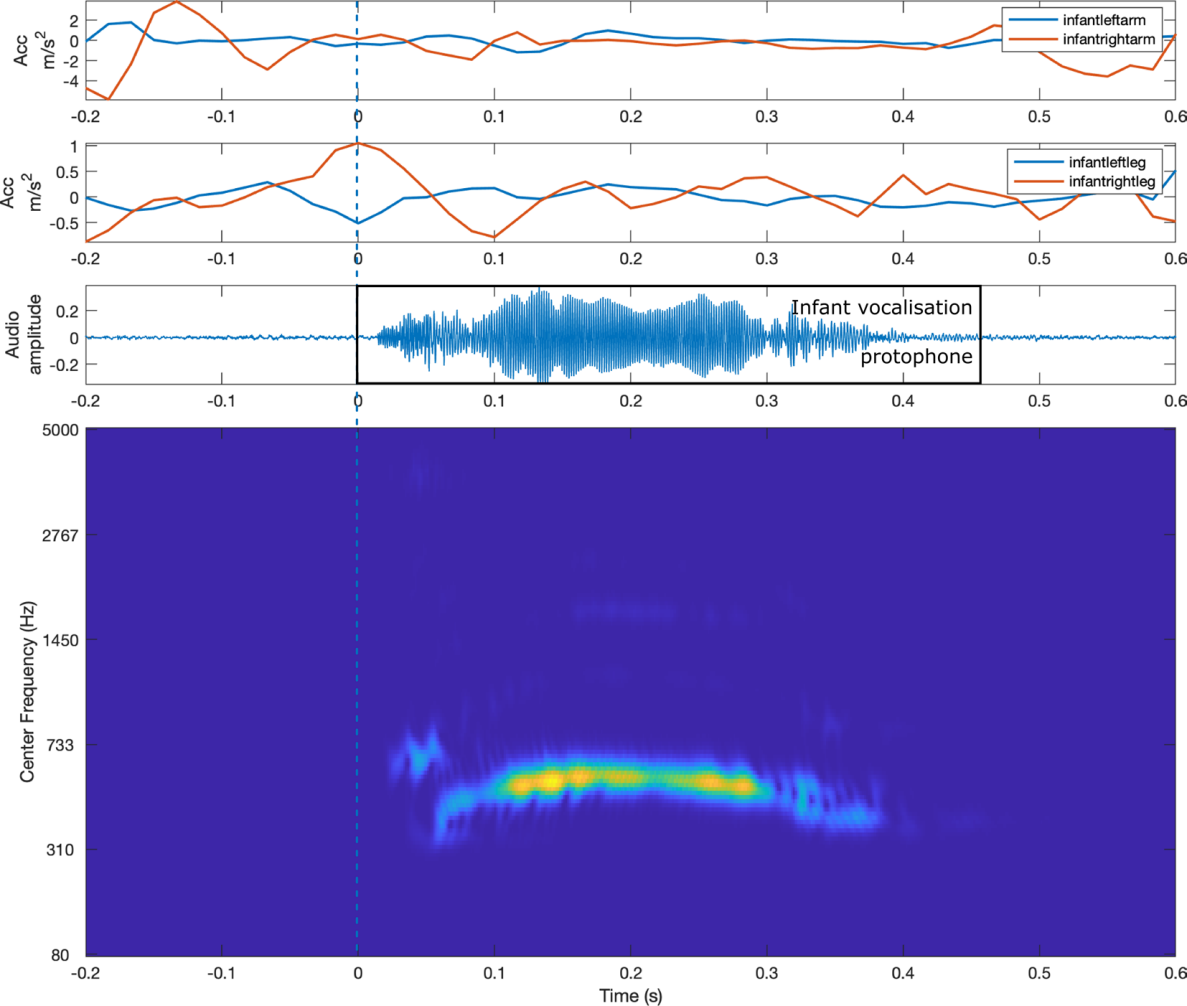
Example of an infant vocalisation event with multimodal signals. The top panel shows the euclidean of 3-axis accelerometer signals from the infant’s arms and legs. The middle panel displays the audio waveform, including the infant’s vocalisation. The bottom panel presents the cochleogram of a vocalisation, illustrating its spectral and temporal structure.

The next step was to investigate changes in wearable signals around the onset of vocalisation, marked as 0 s. Both audio and **Acc** signals were segmented into time windows ranging from −3.5 s to 5 s (see Fig. 3 for signal example). Because of the nature of IMU accelerometer signals, unconventional baseline correction techniques were applied based on^46^. For each segment, we calculated the mean value of the upper and lower envelopes within the 3.5 to 5-s range and subtracted this mean value from the corresponding segment. Next, we recalculated the analytic envelope using a 12-tap FIR filter from the baselined signal, a standard technique for various types of accelerometer data^47^. We utilised the envelope function with the ‘analytic’ parameter (see Fig. 5), which returns the analytic envelope via a 12-tap FIR filter that preserves phase^48^, thus accommodating the sinusoidal nature of IMU signals.

**Fig. 4:**
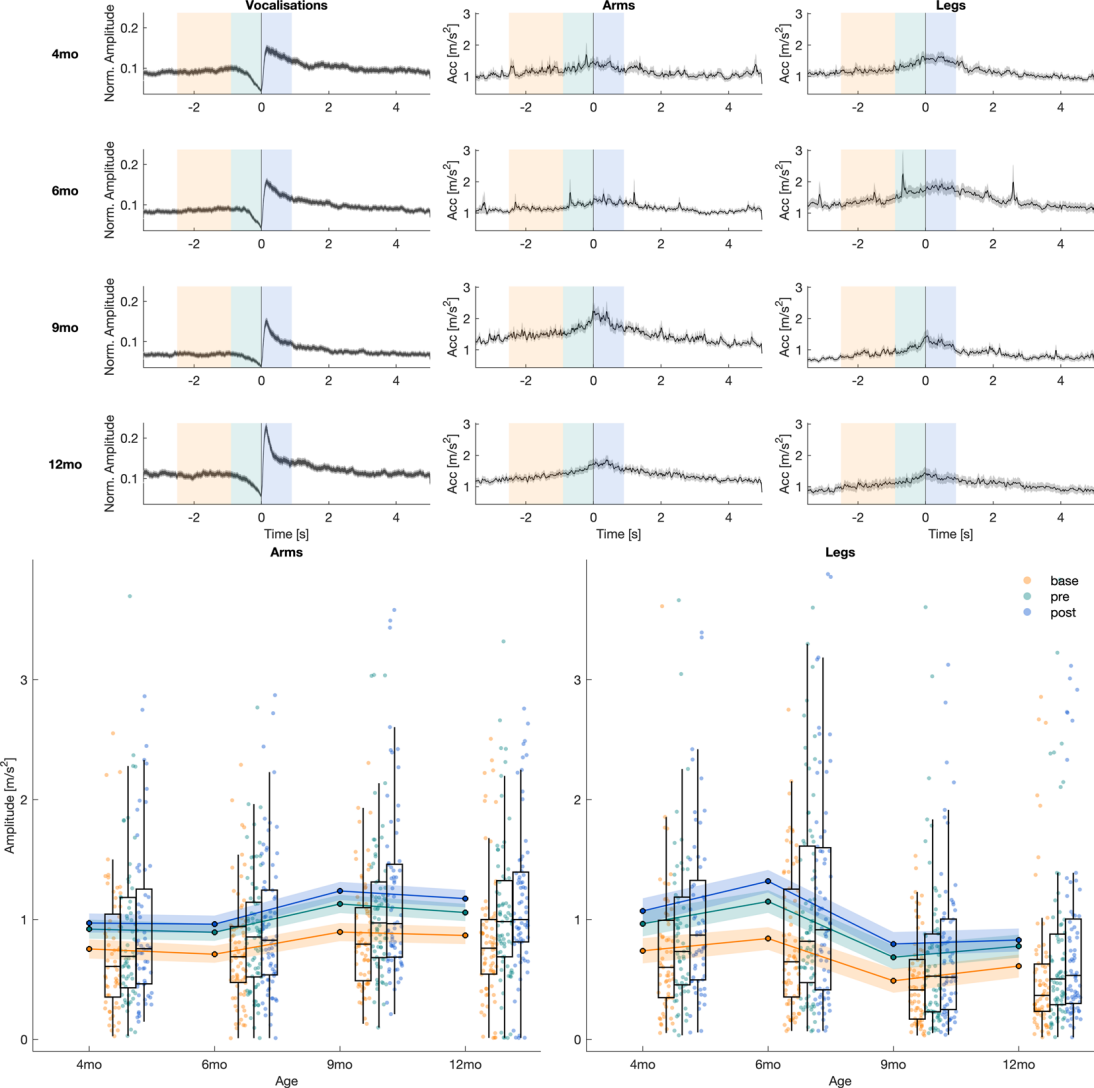
Temporal dynamics of infant vocalisation and limb movement. Illustration of temporal dynamics of infant vocalisation and limb movement during book-sharing tasks from 4 to 12 months of age. The plotted data show averaged envelope signals for vocalisation and movements of the infant’s arms and legs. The shaded grey area represents the standard error. The bottom plots show boxplots of mean values across time points and time windows for each limb separately. For each child, median values were first calculated within each time window, and then these medians were averaged across children. Scatter plots show the mean values from individual children at each time point for limb movement data. Each plot displays the overall mean (solid line) and 95% confidence intervals (shaded area) estimated from a full factorial linear mixed-effects model. Soft hues (blue, yellow, and teal) represent different time windows, highlighting temporal variations in task performance.

Vocalisation signals shown in Fig. 4 represent the average of the upper envelope of individual vocalisations, calculated across all participants. These signals are based on audio recordings captured from the entire room. A decrease in the signal indicates a silent period immediately preceding the onset of vocalisation, while a shift in peak amplitude occurs around the vocalisation onset. Finally, based on the magnitude of change observed in the envelope of the averaged audio signal, three time windows were selected for analyses of acceleration data from IMUs (see Fig. 4, Plot panels, indicated by the black line): “baseline” from −2.5 s to −0.9 s, “pre” (preceding vocalisation onset) from −0.9 s to 0 s, and “during” vocalisation from 0 s to 0.9 s. The inflection point of the averaged audio signal visible across all age groups determined the value of +/- 0.9 s. Within the chosen time window and for each limb, the median of the sensor data was calculated separately for the left and right sides. The median values obtained for each side were averaged to provide a single representative value for each limb and time window.

### Statistical analysis

All the statistical analysis was conducted in the R environment^49^; *lme4* package^50^; *emmeans* package^51^; and *lmerTest* package^52^. The dependent measure for a single vocalisation for a given infant, time window, and limb type at a specific age is calculated as the mean of the medians of the signals from the left and right limbs. Prior statistical analysis confirmed no significant differences between sides at any time point, justifying their averaging for the present analyses.

Linear mixed models were developed to analyse median acceleration, using three predictor variables (Time_Point, Time_Window, and Limb). We selected linear mixed-effects models because the data have a repeated-measures, hierarchical structure, with multiple observations nested within infants across time points. This approach provides greater flexibility than traditional repeated-measures ANOVA in handling missing values, and it was preferred over generalized estimating equations (GEE), due to easier rigorous evaluation of competing models thanks to the possibility of directly applying model comparison criteria (AIC, BIC, log-likelihood).

Four models were constructed, as described in Eqs. (9) through (12). Models described by Eqs. (9—11) included random intercepts for each infant and random slopes for the effect of Limb within each infant, accounting for the correlation between these random effects. In the model developed for data corrected for baseline (Pre-to_Baseline), random slopes were no longer statistically significant (see Table S2). As shown in Eq. (12), the model included only a random intercept for each infant.

Global model for triple interaction Time_Point, Time_Window and Limb. We defined and tested eight different models, evaluating them using Akaike’s Information Criterion, Bayesian Information Criterion, and the log-likelihood (see Table S3). The model with the highest log-likelihood, which included a triple interaction, was identified as the best model. This was determined by comparing it with modified models and performing a likelihood ratio chi-square test (χ2).

$$median = {\beta }_{0} + {\beta }_{1}{Limb}+ {\beta }_{2}Time\_Window + {\beta }_{3}{Time\_Point}+$$

$$+ {\beta }_{4}Tim{e}_{Point}:Limb + {\beta }_{5}Tim{e}_{Window}:Limb + {\beta }_{6}Tim{e}_{Point}:Tim{e}_{Window}+$$

2 $${\beta }_{7}Time\_Window:Time\_Point :Limb + (1+Limb | infant) + \varepsilon$$

The coactivation between Time_Window and Time_Point median values of motor activity around vocalisation.

$$median = {\beta }_{0} +Time\_Window+ {\beta }_{3}{Time\_Point}+{\beta }_{6}Time\_Point :Time\_Window$$

3 $$(1+Limb | infant) + \varepsilon$$

The interaction between Time_Point and Limb on the pre/during-vocalisation median values of pre/during-vocalisation motor activity around vocalisation.

$$media{n}_{pre/during/base} = {\beta }_{0} + {\beta }_{3}{Time\_Point}+ {\beta }_{1}Limb +$$

4 $${\beta }_{5}Time\_Point : Limb+ (1 + Limb | infant) + \varepsilon$$

The difference in coactivation relative to Time_Point and Limb type is reflected in the variation of motor activity around vocalisation between the pre/during-vocalisation periods and the baseline.

$$media{n}_{diffpre/diffduring} = {\beta }_{0} +{\beta }_{1}Limb + {\beta }_{3}{Time\_Point}+$$

5 $${\beta }_{5}Time\_Point : Limb +(1 | infant)+ \varepsilon$$

where, for all models:

- $median$ represents the median acceleration of the left and right limbs.
- ${\beta }_{0}$ is the intercept.
- ${\beta }_{1}$, ${\beta }_{2}$, ${\beta }_{3}$​, ${\beta }_{4}$, ${\beta }_{5}$​, ${\beta }_{6}$, ${\beta }_{7}$ are the coefficients for $Time\_Point$, $Limb$, $Tme\_Window$, and the interaction between $Limb$ and $Time\_Window$, $Time\_Point$ and $Limb$, $Time\_Point$ and $Time\_Window$, and $Time\_Window$, $Time\_Point$ and $Limb$ respectively, values of the $\beta$ s differ between models.
- $(1 + Limb | infant)$ specifies that each infant has an intercept and slope for the Limb, allowing both parameters to vary individually.
- $(1|infant)$ accounts for random differences in intercepts for each infant.
- $\varepsilon$ represents the error term.

To obtain a comprehensive evaluation of both fixed and random effects, we assessed the significance of interactions using *ANOVA* (*type* = ’II’, *ddf* = ’Satterthwaite’, which helps to understand the significance of each fixed effect in the model), *ranova* (which evaluates the significance of the random effects by comparing models with and without specific random effects), and examined specific contrasts with post-hoc tests using the *emmeans* package. Finally, pairwise comparisons were performed for selected means and adjusted using the Bonferroni correction with Cohen’s *f* reported to quantify effect sizes and the strength of the model.

### Transparency and openness

The data supporting the findings (raw data with annotation files, as well as the preprocessed, filtered, and epoched data for each participant around the onset of vocalisations) will be available upon request from the corresponding authors following an embargo period of 12 months from the date of the publication to allow for the finalisation of the ongoing longitudinal project. In accordance with the European General Data Protection Regulation (GDPR), any personally identifiable data, such as videos and audio recordings, will not be shared. Only anonymised and non-identifiable data will be accessible. The analytic code is publicly accessible at the following URL: https://github.com/JDudaGolawska/Movin-Motor-Vocal-Paper-.git

## Results

Infants’ vocalisations produced during every testing session (at 4, 6, 9 and 12 months of age) were manually annotated. The average number of vocalisations across time points was *M* = 2132, *SD* = 280, with an average number of vocalisations per visit T1 = 34, T2 = 33, T3 = 25, T4 = 31 (see Table 1 for details). The onset of each vocalisation was acoustically identified to define three time windows: baseline (–2.5 to –0.9 s), pre- (–0.9 to 0 s), and during-vocalisation (0 to 0.9 s). For each window and limb, median sensor values were calculated separately for left and right sides and then averaged, yielding a single value per limb and time window at 4, 6, 9, and 12 months (see Methods: Movement Data Pre-processing for details).

### Full factorial model

In the first step, we conducted a full factorial (*Time_Window* x *Limb* x *Time_Point*) linear mixed-model analysis (*see Eq. *1 and Supporting Information Table S1 for full description) to test (1) whether limbs were coupled with the onset of a vocalisation (i.e. main effect of time window) (2) whether arms were more coupled than legs (i.e. main effect of limb); and (3) whether this coupling changed with age (i.e. main effect of time point). The model showed a triple interaction, indicating a complex developmental process (see Fig. 4). The explained variance was modest for fixed effects (Marginal R^2^ = 0.019) but substantially higher when accounting for random effects (Conditional R^2^ = 0.247). To explain each of the interaction effects, we conducted simpler, two-way linear mixed models.

### Coupling between limb movements and vocalisations across all time points

In the second step, we tested whether limbs are coupled with the onset of a vocalisation, similarly across all time points. We fitted a linear mixed-effects model (*Time_Window x Time_Point*) to investigate the interaction of time window (baseline, pre-vocalisation, during-vocalisation) specific time points (4, 6, 9, and 12 months, see Eq. 2).

We found (see Table 2) a significant main effect of the time window (*p* < 0.001, Cohen’s *f* = 0.103), indicating that median acceleration significantly increased across the time windows. However, there was no significant main effect of time point (*p* = 0.761) and no significant time window x time point interaction (*p* = 0.115).

**Table 2:** ANOVA and post-hoc results for the Median Acceleration model.

ANOVA Table (Type II)									
Predictor	Sum Sq	Mean Sq	Num DF	Den DF	F-val	p-val	η^2^p	CI [0.95]	Cohen f
**Time Window**	**836.18**	**418.09**	**2**	**50,451.66**	**274.78**	** <.001**	**0.008**	**0.007–1**	**0.103**
Time Point	1.78	0.59	3	272.95	0.39	.761	0.004	0.000–1	0.065
Time Window: Time Point	15.58	2.60	6	50,451.66	1.71	0.115	0.000	0.000–1	0.014

**ANOVA-Like Table for Random-Effects**									
**Model Component**	npar	LogLik	AIC	LRT	Df	p-val			
Without random eff.	16	−83,907.41	167,846.81	-	-	-			
**(1 + Limb | infant)**	**14**	**−85,744.69**	**171,517.37**	**3674.556**	**2**	** <.001**			

**Post-hoc Comparisons (Emmeans)**									
Contrast	Estimate [m/s^2^]	SE [m/s^2^]	df	t	p-val	B.corr. p-val			Cohen f
**base—pre**	**−0.21**	**0.01**	**50,448.45**	**−15.51**	** <.001**	** <.001**			**0.169**
**base—during**	**−0.31**	**0.01**	**50,448.45**	**−22.75**	** <.001**	** <.001**			**0.248**
**pre—during**	**−0.10**	**0.01**	**50,448.45**	**−7.24**	** <.001**	** <.001**			**0.079**

Comprehensive results table: ANOVA, random effects, and post-hoc comparisons for the model *Median Acceleration* ~ *Time_Window x Time_Point* + *(1* + *Limb | infant).*

Post-hoc comparisons confirmed that limb acceleration significantly increased between the baseline, when infants were not producing any speech-like sounds, and the two time windows around the onset of a vocalisation (pre-vocalisation vs baseline and during-vocalisation vs baseline, both *ps* < 0.001, Cohen’s *f* = 0.169 and 0.248, respectively). Additionally, there was a significant increase in median acceleration from the pre-vocalisation to the during-vocalisation period (*p* < 0.001, Cohen’s *f* = 0.079), indicating that limb acceleration increased further within 900 ms after the onset of a vocalisation.

Overall, this analysis demonstrated that limb movements (both arms and legs) were consistently coupled with vocalisations across all time points (4–12 months of age). We found that arm and leg movements considerably increase when infants are about to produce a vocalisation. These movements increased even further when that vocalisation began.

### Developmental changes in arm vs. leg coactivations during vocalisations

**Fig. 5:**
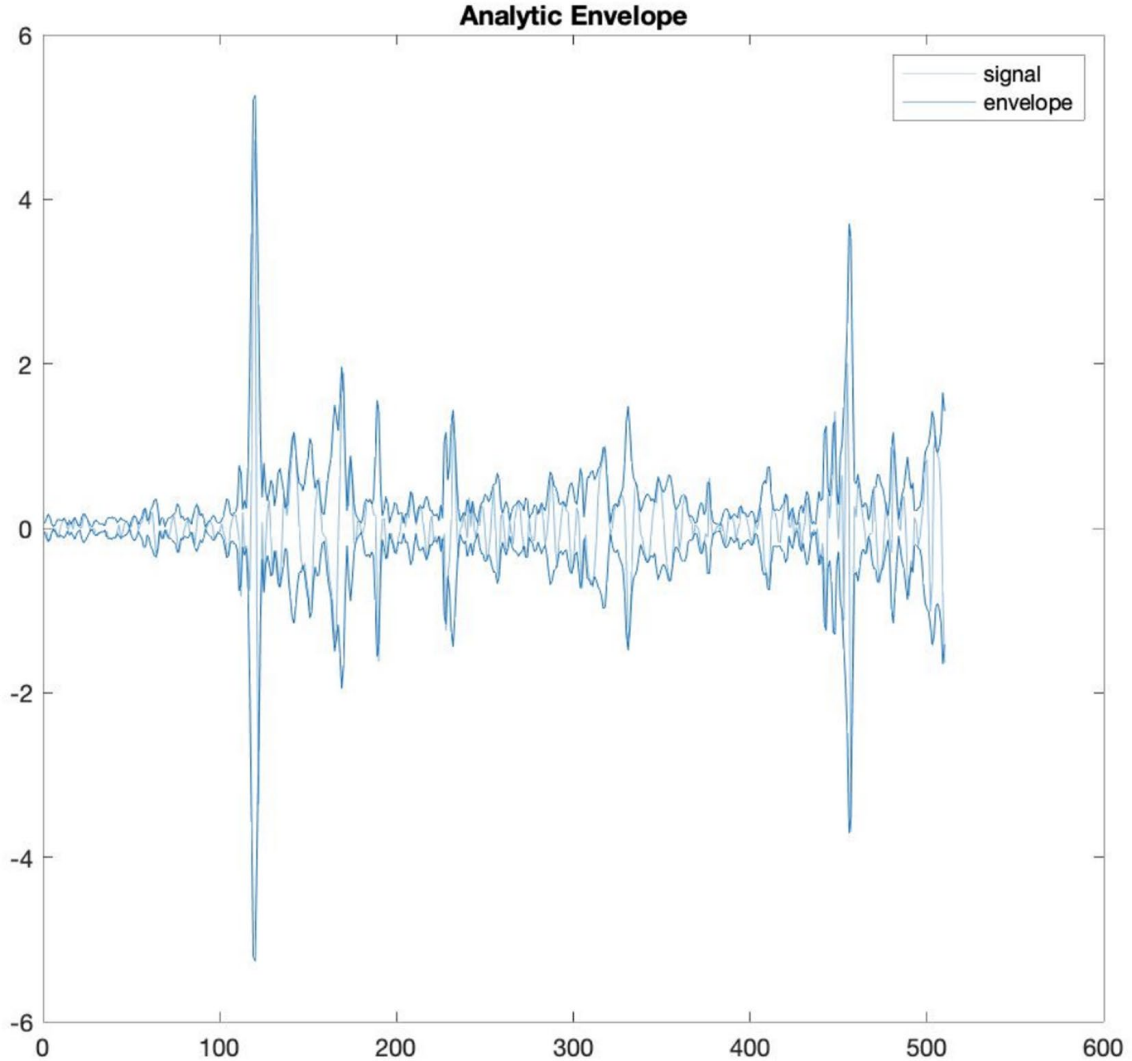
Top and bottom envelope of Acc signal. The figure presents three signals: the upper envelope (top envelope) and lower envelope (bottom envelope) of the Acceleration (Acc) signal, shown as bold lines, and the original Acc signal with the mean of the two envelopes subtracted, shown as a lighter line. This decomposition allows visualisation of the relative movement dynamics after removing the average trend captured by the envelopes.

In the previous step, we demonstrated motor-vocal coupling across infancy. Next, we investigated developmental changes in this coupling, to see whether arm and leg movements follow the same developmental pattern. Since previous analysis showed that coupling of limbs before and after vocalisation onset differed, we investigated the age-related effects separately in the pre- and during-vocalisation time windows (*Time_Point x Limb;* see Table 3, Eq. 3). There were no main effects on either the limb or the time point. However, there was a significant interaction of limb x time point for both pre- and during vocalisation (*ps* < 0.001, Cohen’s *f* = 0.343 and 0.374, respectively). In both time windows at 4 months of age, there was no difference in the level of coupling between arms and legs (both *ps* = 1). At 6 months, legs exhibited significantly higher activity than arms (pre-: *p* = 0.025, Cohen’s *f* = 0.202; during-vocalisation: *p* = 0.003, Cohen’s f = 0.260). By contrast, from 9 months (both *ps* < 0.001, pre- Cohen’s *f* = 0.348 and during- vocalisation 0.328) through to 12 months of age (pre-: *p* = 0.011, Cohen’s *f* = 0.221; during-vocalisation: *p* = 0.003, Cohen’s *f* = 0.258), this trend reversed, with arms showing significantly higher activity compared to legs just before and just after the onset of vocalisation (Fig. 5).

**Table 3:** ANOVA and post-hoc results for the Pre- and During Vocalisation Median Acceleration model.

ANOVA Table (Type II) Pre-vocalisation									
Predictor	Sum Sq	Mean Sq	Num DF	Den DF	F-val	p-val	η^2^p	CI [0.95]	Cohen f
Time Point	2.17	0.72	3	271.94	0.45	.720	0.005	0.000–1	0.070
Limb	4.54	4.54	1	272.00	2.82	.094	0.016	0.001–1	0.129
**Time Point:Limb**	**51.96**	**17.32**	**3**	**273.52**	**10.73**	** <.001**	**0.105**	**0.049–1**	**0.343**

**ANOVA-Like Table for Random-Effects Pre-vocalisation**									
**Model Component**	npar	LogLik	AIC	LRT	Df	p-val			
Without random eff.	12	−28,729.59	57,483.17	-	-	-			
**(1 + Limb | infant)**	**10**	**−29,136.88**	**58,293.76**	**814.59**	**2**	** <.001**			

**Post-hoc Comparisons (Emmeans) Pre-vocalisation**									
Contrast	Estimate [m/s^2^]	SE [m/s^2^]	df	t	p-val	B.corr. p-val			Cohen f
mo4 leg—mo4 arm	0.05	0.11	261.41	0.44	.660	1			0.037
**mo6 leg—mo6 arm**	**0.26**	**0.09**	**252.76**	**2.76**	**.006**	**.025**			**0.202**
**mo9 leg—mo9 arm**	**−0.44**	**0.10**	**274.71**	**−4.45**	** <.001**	** <.001**			**0.348**
**mo12 leg—mo12 arm**	**−0.28**	**0.09**	**246.87**	**−3.01**	**.003**	**.011**			**0.221**

**ANOVA Table (Type II) During-vocalisation**									
Predictor	Sum Sq	Mean Sq	Num DF	Den DF	F-val	p-val	η^2^p	CI [0.95]	Cohen f
Time Point	2.75	0.92	3	274.62	0.48	.694	0.005	0.000–1	0.073
Limb	1.92	1.92	1	271.78	01.01	.316	0.008	0.000–1	0.088
**Time Point:Limb**	**72.39**	**24.13**	**3**	**273.20**	**12.73**	** <.001**	**0.123**	**0.063–1**	**0.374**

**ANOVA-Like Table for Random-Effects During-vocalisation**									
**Model Component**	npar	LogLik	AIC	LRT	Df	p-val			
Without random eff.	12	−30,115.78	60,255.57	-	-	-			
**(1 + Limb | infant)**	**10**	**−30,559.48**	**61,138.95**	**887.39**	**2**	** <.001**			

**Post-hoc Comparisons (Emmeans) During-vocalisation**									
Contrast	Estimate [m/s^2^]	SE [m/s^2^]	df	t	p-val	B.corr. p-val			Cohen f
mo4 leg—mo4 arm	0.13	0.12	263.79	01.05	.295	1			0.091
**mo6 leg—mo6 arm**	**0.36**	**0.11**	**254.25**	**3.41**	**.001**	**.003**			**0.260**
**mo9 leg—mo9 arm**	**−0.45**	**0.11**	**274.98**	**−4.03**	** <.001**	** <.001**			**0.328**
**mo12 leg—mo12 arm**	**−0.35**	**0.11**	**247.83**	**−3.38**	**.001**	**.003**			**0.258**

Comprehensive results table: ANOVA, random effects, and post-hoc comparisons for the model *Median Acceleration Pre/During* ~ *Time_Point x Limb* + *(1* + *Limb | infant).*

\\\\

**Fig. 6:**
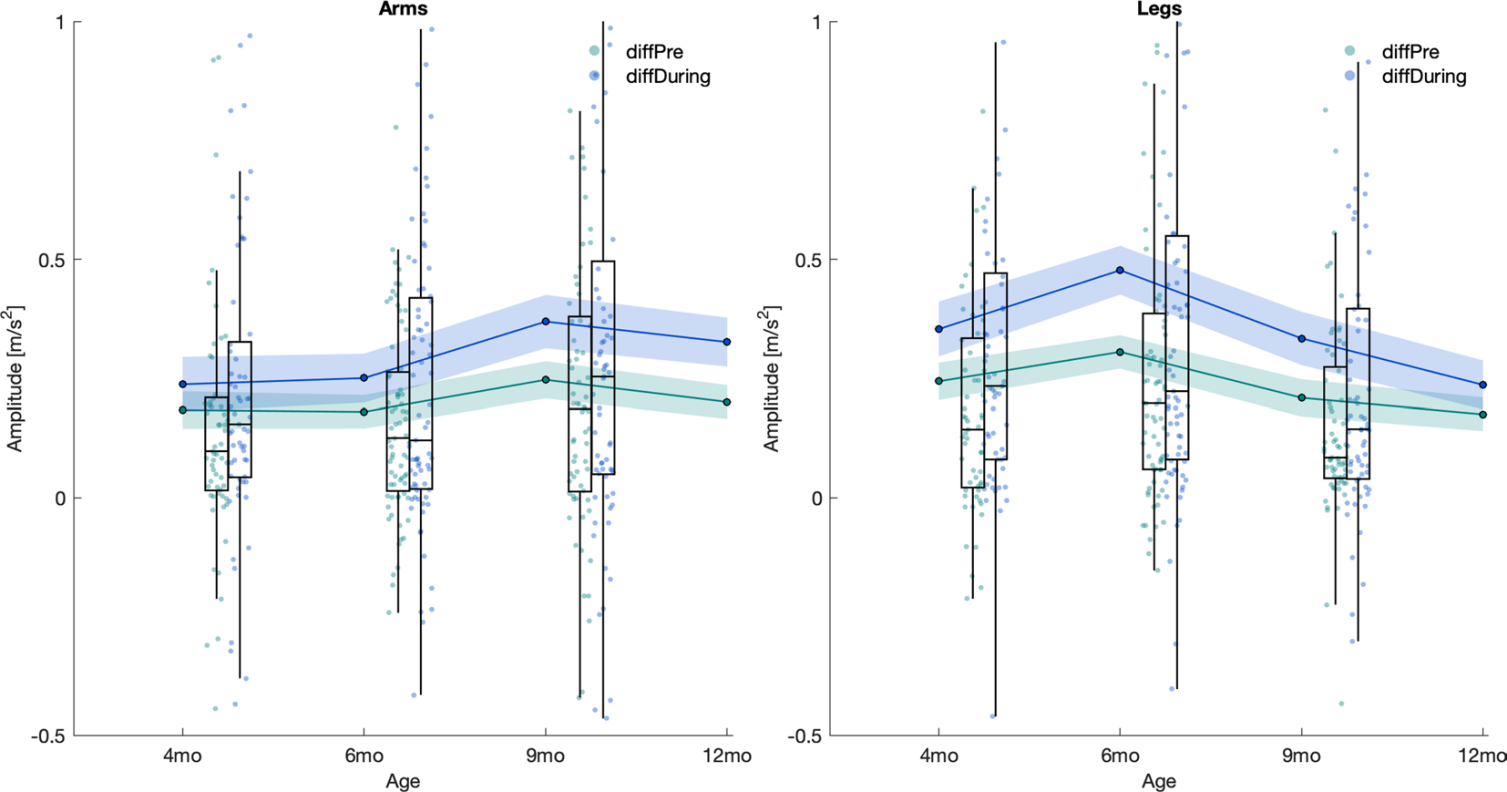
Distribution of arm and leg movements around vocalisation onset corrected for baseline. Illustration of the difference in distribution between Pre/During-vocalisation vs baseline of arm and leg movements across different time points surrounding the onset of vocalisation. The subplots show boxplots of mean values across time points and time windows for Arms (left) and Legs (right) separately. For each child, median values were first calculated within each time window, and then these medians were averaged across children. Scatter plots show the mean values from individual children at each time point for limb movement data. Each plot displays the overall mean (solid line) and 95% confidence intervals (shaded area) estimated from a *Median Acceleration Pre/During-to-Baseline* ~ *Time_Point x Limb* + *(1 | infant)* linear mixed-effects model.

Previous analysis revealed that limb activity coupled with vocalisations may undergo developmental changes. The relative magnitude of arm vs leg movement appeared to change across time points. It was not clear whether these changes were affected by likely baseline differences in arm vs leg activity, due to, e.g. the acquisition of new gross motor skills. To test this we conducted a control analysis (*Time_Point x Limb*) on the acceleration from the baseline time window alone (see Table 4, Eq. 4), which confirmed this developmental trend (see Fig. 6).

**Table 4:** ANOVA and post-hoc results for the Baseline Median Acceleration model.

ANOVA Table (Type II) Baseline									
Predictor	Sum Sq	Mean Sq	Num DF	Den DF	F-val	p-val	ŋ^2^p	CI [0.95]	Cohen f
Time Point	2.145	0.715	3	275.245	0.686	.561	0.007	0.000–1	0.086
**Limb**	**9.362**	**9.362**	**1**	**279.052**	**8.978**	**.003**	**0.041**	**0.012–1**	**0.207**
**Time Point:Limb**	**34.823**	**11.608**	**3**	**280.835**	**11.132**	** <.001**	**0.106**	**0.051–1**	**0.345**

**ANOVA-Like Table for Random-Effects Baseline**									
**Model Component**	npar	LogLik	AIC	LRT	Df	p-val			
Without random eff.	12	−24,987.4	49,998.8	-	-	-			
**(1 + Limb | infant)**	**10**	**−25,335.61**	**50,691.22**	**696.43**	**2**	** <.001**			

**Post-hoc Comparisons (Emmeans) Baseline**									
Contrast	Estimate [m/s^2^]	SE [m/s^2^]	df	t	p-val	B.corr. p-val			Cohen f
**leg—arm**	**0.13**	**0.04**	**256.04**	**−3.47**	**.001**	**.001**			**0.124**
mo4 leg—mo6 leg	−0.12	0.11	269.40	−1.06	.29	1			0.116
mo4 leg—mo4 arm	0.00	0.08	255.69	0.03	.97	1			0.003
**mo6 leg—mo9 leg**	**0.38**	**0.11**	**269.66**	**3.53**	**.001**	**.005**			**0.374**
mo6 leg—mo6 arm	0.14	0.07	249.15	1.96	.05	.508			0.133
mo9 leg—mo12 leg	−0.13	0.11	265.73	−1.15	.25	1			0.122
**mo9 leg—mo9 arm**	**−0.39**	**0.07**	**273.70**	**−5.33**	** <.001**	** <.001**			**0.386**
**mo12 leg—mo12 arm**	**−0.25**	**0.07**	**244.53**	**−3.61**	** <.001**	**.004**			**0.244**
mo4 arm—mo6 arm	0.01	0.09	261.61	0.16	.87	1			0.014
mo6 arm—mo9 arm	−0.15	0.08	266.31	−1.77	.08	.783			0.144
mo9 arm—mo12 arm	0.02	0.08	262.99	0.25	.8	1			0.020

Comprehensive results table: ANOVA and random effects for model Median *Acceleration Baseline* ~ *Time_Point x Limb* + *(1* + *Limb | infant).*

There was a main effect of limb (*p* = 0.003, Cohen’s *f* = 0.207) and a significant limb × time point interaction (*p* < 0.001, Cohen’s *f* = 0.345).

The significant main effect of the limb indicated that leg movements were overall greater than arm movements (*p* = 0.003, Cohen’s *f* = 0.207). However, the difference between leg and arm movements varied across the four time windows, with the magnitude and direction of this difference changing over time. Baseline acceleration was lower for legs than arms at 9 and 12 months of age (both *ps* < 0.005, Cohen’s *f* = 0.386 and 0.244, respectively), while it remained comparable at both 4 and 6 months of age (both *ps* > 0.5). Leg acceleration alone decreased significantly between 6 and 9 months of age (*p* = 0.005, Cohen’s *f* = 0.374).

Since the baseline level of limb activity also changed with age, the last control analysis tested its effects on our main findings. We subtracted the baseline level of acceleration from acceleration in the pre- and during-vocalisation time windows. These differential (pre-to-baseline and during-to-baseline acceleration) measures were used as dependent variables in two control models, which analysed arms vs leg activity related to vocalisations across time points (*Time_Point x Limb*, see Table 5 and Eq. 5). After adjusting for baseline, analyses revealed that leg acceleration in the during-vocalisation time windows was significantly higher than arm acceleration (*p* = 0.022, Cohen’s *f* = 0.035). Generally, a comparison of limb activation revealed that activation levels at time points before or after vocalisation were similar, except at 6 months, where both the pre- (*p* = 0.005, Cohen’s *f* = 0.100) and during-vocalisation (*p* < 0.001, Cohen’s *f* = 0.148) time windows showed significantly higher activation in the legs compared to the arms.

**Table 5:** ANOVA and post-hoc results for the Pre- and During-to-Baseline Median Acceleration model.

ANOVA Table (Type II) Pre-to-Baseline									
Predictor	Sum Sq	Mean Sq	Num DF	Den DF	F-val	p-val	ŋ^2^p	CI [0.95]	Cohen f
Time Point	2.85	0.95	3	206.38	0.61	.611	0.009	0.000–1	0.094
Limb	5.36	5.36	1	16,724.33	3.43	.064	0.00	0.000–1	0.012
**Time Point:Limb**	**19.22**	**6.41**	**3**	**16,724.33**	**4.10**	**.006**	**0.001**	**0.000–1**	**0.027**

**ANOVA-Like Table for Random-Effects Pre-to-Baseline**									
**Model Component**	npar	LogLik	AIC	LRT	Df	p-val			
Without random eff.	10	−28,039.03	56,098.06						
**(1 | infant)**	**9**	**−28,093.80**	**56,205.61**	**109.55**	**1**	** <.001**			

**Post-hoc Comparisons (Emmeans) Pre-to-Baseline**									
Contrast	Estimate [m/s^2^]	SE [m/s^2^]	df	t	p-val	B.corr. p-val			Cohen f
mo4 leg—mo6 leg	−0.06	0.05	395.21	−1.17	.241	1			0.049
mo4 leg—mo4 arm	0.06	0.04	16,756.25	1.55	.121	1			0.048
mo6 leg—mo9 leg	0.10	0.05	475.47	1.84	.07	.668			0.077
**mo6 leg—mo6 arm**	**0.13**	**0.04**	**16,756.25**	**3.47**	**.001**	**.005**			**0.100**
mo9 leg—mo12 leg	0.03	0.05	490.65	0.65	.514	1			0.027
mo9 leg—mo9 arm	−0.04	0.04	16,756.25	−0.90	.370	1			0.030
mo12 leg—mo12 arm	−0.03	0.04	16,756.25	−0.69	.493	1			0.020
mo4 arm—mo6 arm	0.00	0.05	395.21	0.07	.944	1			0.003
mo6 arm—mo9 arm	−0.07	0.05	475.47	−1.28	.203	1			0.053
mo9 arm—mo12 arm	0.05	0.05	490.65	0.88	.377	1			0.037

**ANOVA Table (Type II) During-to-Baseline**									
Predictor	Sum Sq	Mean Sq	Num DF	Den DF	F-val	p-val	ŋ^2^p	CI [0.95]	Cohen f
Time Point	5.20	1.73	3	242.52	0.74	.531	0.009	0.000–1	0.096
**Limb**	**16.00**	**16.00**	**1**	**16,735.32**	**6.81**	**.009**	**0.000**	**0.000–1**	**0.018**
**Time Point:Limb**	**69.74**	**23.25**	**3**	**16,735.32**	**9.90**	** <.001**	**0.002**	**0.001–1**	**0.042**

**ANOVA-Like Table for Random-Effects During-to-Baseline**									
**Model Component**	npar	LogLik	AIC	LRT	Df	p-val			
Without random eff.	10	−31,550.27	63,120.54						
**(1 | infant)**	**9**	**−31,710.33**	**63,438.65**	**320.11**	**1**	** <.001**			

**Post-hoc Comparisons (Emmeans) During-to-Baseline**									
Contrast	Estimate [m/s^2^]	SE [m/s^2^]	df	t	p-val	B.corr. p-val			Cohen f
**leg—am**	**0.05**	**0.02**	**16,742.43**	**2.3**	**0.022**	**0.022**			**0.035**
mo4 leg—mo6 leg	−0.12	0.08	359.78	−1.60	.110	1			0.081
mo4 leg—mo4 arm	0.12	0.05	16,742.43	2.45	.014	.143			0.076
mo6 leg—mo9 leg	0.14	0.08	402.46	1.89	.060	.597			0.094
**mo6 leg—mo6 arm**	**0.23**	**0.04**	**16,742.43**	**5.12**	** <.001**	** <.001**			**0.148**
mo9 leg—mo12 leg	0.10	0.08	405.87	1.28	.200	1			0.064
mo9 leg—mo9 arm	−0.04	0.05	16,742.43	−0.69	.493	1			0.023
mo12 leg—mo12 arm	−0.09	0.05	16,742.43	−1.96	.0495	.495			0.059
mo4 arm—mo6 arm	−0.01	0.08	359.78	−0.17	.866	1			0.008
mo6 arm—mo9 arm	−0.12	0.08	402.46	−1.56	.119	1			0.077
mo9 arm—mo12 arm	0.04	0.08	405.87	0.57	.0.571	1			0.028

Comprehensive results table: ANOVA, random effects, and post-hoc comparisons for model *Median Acceleration Pre/During-to-Baseline* ~ *Time_Point x Limb* + *(1 | infant).*

## Discussion

The execution of motor actions underpins vocal production – uttering a single syllable involves over 70 muscles in the mouth and tongue as well as respiratory, laryngeal, and pharyngeal systems^53,54^. Other body parts seem to also be co-activated during vocal production^23^. Yet, this process has been largely overlooked as the foundation for prelinguistic speech development. We addressed this considerable gap in knowledge by investigating longitudinal changes in motor-vocal coupling between infants’ limb movements and vocalisations. We measured their motor behaviour with wearable motion trackers and annotated vocal production during play with caregivers. We then analysed changes in the level of acceleration of limb movements around the onset of vocalisation in relation to a preceding baseline period when no vocalisation was produced. We found that limb movements are coupled with vocalisation onsets at all tested time points (4, 6, 9 and 12 months) across the first year of life. However, we also showed that this motor-vocal coupling may undergo a reorganisation in infancy.

Our results indicate a consistent coupling of vocal production with both arm and leg movements in typical development throughout 4–12 months of age. Of particular interest is the strong coupling between all limbs – arms as well as legs – and vocalisations from as early as 4 months of age. In contrast to previous research, we have not constrained either the infant’s body position or the infant-caregiver dyadic positioning, which allowed us to show that this coupling is not restricted to a single body position, as shown by Borjon et al.^23^ during face-to-face tabletop play from 9 months of age onwards. The coupling is also present in pre-sitting infants and infants that do not yet produce more advanced vocalisations, such as reduplicated babbling. This is an important finding, as previous literature suggested that arm movements co-occur mostly with reduplicated babbling due to the shared rhythmic character of both types of activities^20^. In contrast to Iverson & Fagan^20^ we have not examined motor-vocal coupling in relation to different types of vocalisations, thus, future research should look into more fine-grained comparisons to better understand this developmental process. Altogether, here we demonstrate that the coupling between limbs and vocal production is a general phenomenon that occurs consistently in the same individuals throughout most of infancy.

The results showing motor-vocal coupling across infancy are in line with the theoretical proposal by Pouw and Fuchs^5^. They suggested that vocal production is inherently coupled with body movements through links with the respiratory system^48^. In their proposal body positioning and various types of body movements constrain sound production in humans and other species. However, previous results on adult humans (and non-human animals) suggested a rather weak coupling between limb movements and vocalisations (see also an extensive review on the sound-movement coupling across species in^55^). In contrast, our longitudinal results from the developmental perspective indicate a strong coupling present from 4 months of age, which does not weaken across the first year of life.

Our finding of the coupling between legs and vocalisations is a novel one. Previous studies of early development showed the coupling of human infants’ arms and heads during vocal production^23^ and vocal-locomotor coordination in infant marmoset monkeys^56^. Interestingly, there are only two, but inconsistent reports on the coupling of human adult leg movements with vocal production in different experimental tasks^14,15^, which highlight that the role of various body parts in vocal production is not well understood across the lifespan. Our findings from the infancy period indicate that the developmental process of learning how to coordinate various effectors (different body parts that seem to play a role in speech production) continues throughout the first year of life and extends beyond it.

In adult humans, the coupling between limb movements and vocal production is evident during gesturing. Hand gestures tightly align with speech production on multiple levels: temporal, semantic, pragmatic, and emotive (see review in^57^), but there is very limited data on developmental trajectories that lead to this alignment (e.g^26,58,59^. However, as recently argued by Karadöller et al.^60^, a multimodal approach to language acquisition that encompasses both speech and gesture is inevitable, as spoken language acquisition consists of learning to coordinate linguistic expressions in both speech and gesture. In a commentary to Karadöller et al., Capirci and Iverson^61^ emphasised the importance of viewing language development in the context of the body in which it is embedded. Our results indicate a clear relationship between vocal production and body movements, with early onset of the coupling between arm movements and vocal production at temporal and biomechanic levels.

Nonetheless, another direction of interpretation of our research findings could be the contingency of both vocalisations and limb movements on infants’ arousal state. Wass et al.^62^ showed that at 12 months of age, infants’ vocalisations were strongly contingent on their arousal state for both cries and speech-like vocalisations. Similar findings were shown by Gustison et al.^56^ for infant marmosets – their vocal-locomotor coordination improved with age and during elevated arousal levels. However, previous research by Oller and colleagues may suggest that changes in arousal level may not fully explain the trajectory of vocal development. Human infant speech-like vocalisations (that were studied in the present study) are produced with positive, negative, or neutral facial affect on different occasions, presenting functional flexibility that is not dependent on arousal level^63,64^. Such speech-like vocalisations vastly outnumber cries that are clearly related to higher arousal and negative affect^65^. Furthermore, human infant vocalisations are far less likely to be interpreted as arousal-related complaints and more likely as vocal play than bonobo infant vocalisations^66^, which calls for caution in extrapolating conclusions across species. Since we have not systematically measured arousal fluctuations in infants in our study, we cannot directly investigate the role of arousal in observed motor-vocal coupling. Thus, future research should address this hypothesis in detail, potentially also including the role of breathing^5,67^, which, again, could change depending on infant arousal level.

In the present dataset, we did not distinguish between unimanual and bimanual actions, and thus cannot address whether more rhythmic, bilateral movements (e.g., flapping or kicking) exert different constraints on vocalisations than unimanual movements or gestures (e.g., pointing or object-directed reaches). This distinction could be intriguing, particularly in light of our prior findings of age-related increasing coherence between the left and the right limb in the same cohort^36^ and theoretical notions that rhythmic gestural “babbling” may scaffold speech production^5,20^. Future studies incorporating fine-grained categorisation of movement types and their rhythmic structure will be crucial to test whether bilateral rhythmic movements differentially support or constrain infant vocalisation. Such work would also align with broader evidence that rhythmic and bilateral gestures form a developmental precursor to speech–gesture coordination in later infancy and childhood^1,53,68^.

In addition to already discussed factors, developmental changes in infants’ own motor skills and social engagement across the first year are likely to shape motor–vocal dynamics. As infants gain new postural abilities and increasingly engage in joint attention and reciprocal interactions, their opportunities for coordinating movement with vocal production expand^1,68–71^. These developmental cascades may amplify or reorganise the coupling patterns we observed, highlighting the need to interpret our results within the broader trajectory of motor and social development.

It should also be noted that the book-sharing context itself may have influenced the observed coupling patterns. Shared reading is known to elicit frequent spontaneous infant vocalizations^34^ as well as variability in caregiver engagement and responsiveness, which can shape infant vocal participation and subsequent language outcomes^72–74^. Although our semi-naturalistic design ensured free social interactions, differences in caregivers’ reading styles or attentional focus may have contributed to variability across dyads and developmental stages. Thus, the generalisability of our findings should be considered in light of this situational context.

General spontaneous coupling of limb movements and vocalisations could potentially occur by chance, as a result of an infant exploring all possible ways in which they can move their body (exploration of degrees of freedom). The body movements can affect vocal production through respiratory interactions, which can result in joint neural regulation of manual and vocal systems^5^. As preliminary findings from a case study by Fuchs et al.^67^ suggest, with increasing age and practice, infant vocalisations appear to develop towards the respiratory-laryngeal coordination observed in adults. The respiratory cycles during vocalisation become more asymmetric over time, with shorter inhalations in relation to the overall cycle. Thus, Fuchs et al.^67^ argue that breathing should be considered another aspect on which first vocalisations and later language are built. Our results showing spontaneous coupling of limbs during vocal production could showcase another aspect of this rapidly developing speech-respiration-gesture system, with the initial spontaneous coupling potentially leading to adult-like gestures closely linked to speech production.

The findings presented here suggest new important directions for research on prelinguistic speech development. First, a need to better understand the involvement of limbs in vocal production by tracking the strength of motor-vocal coupling across various body positions. Body positioning has consequences for freeing or constraining limbs in their supporting or stabilising role, but also for the overall positioning of the vocal tract (Ref^29^, e.g., legs are stabilising the upper body but arms are free to move during unsupported sitting, but, in contrast, arms are constrained, but legs are relatively free to move in the forearm support position while prone). The developmental changes in the strength of coupling between arms and legs at different time points in our data seem to reflect the changes in infants’ preferred body position at a given time point (see^37^ for the distribution of body positions across the first year): with a high coupling of legs at 6 months of age, when they are predominantly prone, to relatively higher coupling of arms at 9 and 12 months, when the sitting becomes the most prevalent position.

However, our finding of legs vs. arms differences in limb acceleration during the baseline period, when no vocalisations were produced, suggests that this is a more general developmental process of freeing the arms from their role in stabilising body position and increasingly using them for other purposes such as object manipulation. Nonetheless, it seems to have important consequences for vocal production and should be investigated further – especially considering that early motor deficits are present in many neurodevelopmental disorders, such as Down syndrome^75^, Williams syndrome^76^, and autism spectrum disorder^21^. Similarly, an atypical pattern of vocal production in infancy was observed in infants at risk of developmental disorders such as autism spectrum disorder, Rett syndrome, and fragile X syndrome (see review in^77^). Thus, understanding the developmental changes in the coupling of limb movements and vocalisations in the general population can help to better address the challenge of tracking atypical developmental cascades and designing early interventions.

Altogether, our results shed new light on the biomechanical underpinnings of prelinguistic vocal development. They suggest new potential mechanisms through which early motor development may enable and facilitate emerging prelinguistic vocal production in infancy. Future speech-language research should aim to combine the measurement of developmental changes in the phonatory, articulatory and respiratory systems (e.g^4,67,78^, together with gross motor development^1^ as well as parental verbal input and responsiveness, that jointly shape advances in communicative development.

## Conclusions

Our longitudinal study for the first time demonstrates the coupling of both arm and leg movements with spontaneous vocal production of speech-like sounds across infancy. The involvement of limbs in speech-like vocalisations is a potential mechanism supporting the early development of prelinguistic vocal production in early infancy. At the same time, this coupling should be interpreted in the context of interactive settings such as book-sharing, which may introduce variability in caregiver responsiveness, as well as developmental changes in infants’ motor, vocal and social abilities across the first year. Together, these findings highlight the importance of considering body movement, context, and caregiver interaction jointly in understanding the foundations of early speech development.

## Supplementary Information

Supplementary Information.

## Data Availability

The data supporting the findings (raw data with annotation files, as well as the preprocessed, filtered, and epoched data for each participant around the onset of vocalisations) will be available upon request from the corresponding authors following an embargo period of 12 months from the date of publication to allow for the finalisation of the ongoing longitudinal project. In accordance with the European General Data Protection Regulation (GDPR), any personally identifiable data, such as videos and audio recordings, will not be shared. Only anonymised and non-identifiable data will be accessible. The analytic code is publicly accessible at the following URL: https://github.com/JDudaGolawska/Movin-Motor-Vocal-Paper-.git
