# Supplementary material for: Vocalisations are coupled with movement of all limbs throughout infancy
Source: Sci Rep. 2025 Dec 29;15:44899. doi: 10.1038/s41598-025-28388-6 (PMC12749638; doi:10.1038/s41598-025-28388-6)
Supplement: Supplementary file 1 — Supplementary Information. [file 41598_2025_28388_MOESM1_ESM.docx]

# **Supplementary Materials**

##### **Table S1**. **Triple interaction model: Median Acceleration ~ Time Point x Time Window x Limb + (1 + Limb | infant).** This model examines the fixed effect estimates and random effect variance for the within-subject factors Time Window (baseline), Limb (leg), and Time Point (4 months) on median Limb acceleration.

| **Fixed Effects** | | | |
| --- | --- | --- | --- |
| Predictor | Estimate [m/s²] | CI (95%) | p-value |
| **(Intercept)** | **0.74** | **0.53 – 0.95** | **<.001** |
| **Time Window [pre]** | **0.23** | **0.15 – 0.30** | **<.001** |
| **Time Window [during]** | **0.33** | **0.26 – 0.41** | **<.001** |
| Limb [arm] | 0.02 | -0.18 – 0.21 | .88 0 |
| Time Point [mo6] | 0.10 | -0.17 – 0.38 | .469 |
| Time Point [mo9] | -0.25 | -0.53 – 0.03 | .084 |
| Time Point [mo12] | -0.13 | -0.41 – 0.15 | .371 |
| Time Window [pre] × Limb [arm] | -0.06 | -0.17 – 0.05 | .266 |
| **Time Window [during] × Limb [arm]** | **-0.12** | **-0.22 – -0.01** | **.031** |
| Time Window [pre] × Time Point [mo6] | 0.08 | -0.02 – 0.19 | .111 |
| **Time Window [during] × Time Point [mo6]** | **0.15** | **0.04 – 0.25** | **.005** |
| Time Window [pre] × Time Point [mo9] | -0.03 | -0.14 – 0.08 | .607 |
| Time Window [during] × Time Point [mo9] | -0.03 | -0.14 – 0.09 | .656 |
| Time Window [pre] × Time Point [mo12] | -0.06 | -0.16 – 0.04 | .260 |
| **Time Window [during] × Time Point [mo12]** | **-0.11** | **-0.22 – -0.01** | **.030** |
| Limb [arm] × Time Point [mo6] | -0.15 | -0.41 – 0.12 | .276 |
| **Limb [arm] × Time Point [mo9]** | **0.39** | **0.12 – 0.66** | **.005** |
| Limb [arm] × Time Point [mo12] | 0.24 | -0.02 – 0.50 | .076 |
| Time Window [pre] × Limb [arm]) × Time Point [mo6] | -0.07 | -0.21 – 0.08 | .376 |
| Time Window [during] × Limb [arm]) × Time Point [mo6] | -0.11 | -0.26 – 0.03 | .134 |
| Time Window [pre] × Limb [arm]) × Time Point [mo9] | 0.10 | -0.06 – 0.25 | .220 |
| Time Window [during] × Limb [arm]) × Time Point [mo9] | 0.15 | -0.00 – 0.31 | .058 |
| Time Window [pre] × Limb [arm]) × Time Point [mo12] | 0.09 | -0.06 – 0.23 | .253 |
| **Time Window [during] × Limb [arm]) × Time Point [mo12]** | **0.21** | **0.06 – 0.35** | **.006** |
| **Random Effects** | | | |
| Component | Variance |  |  |
| Residual (σ²) | 1.52 |  |  |
| Random Intercept (τ₀₀) | 0.60 |  |  |
| Random Slope (τ₁₁) | 0.48 |  |  |
| Correlation (ρ₀₁) | -0.71 |  |  |
| ICC | 0.23 |  |  |
| Number of Infants (N) | 277 |  |  |
| Observations | 51 000 |  |  |
| **Marginal R²** | **0.019** |  |  |
| **Conditional R²** | **0.247** |  |  |

#####

##### **Table S2 Ranova Results: Model with Limb as Random Slope.** Evaluation of model with 'Limb' as a random slope: Median Acceleration Pre-to-Baseline ~ Time Point x Limb + (1 + Limb | infant) using the *ranova* function**.**

| **ANOVA-Like Table for Random-Effects Pre-to-Baseline** | | | | | | |
| --- | --- | --- | --- | --- | --- | --- |
| **Model Component** | npar | LogLik | AIC | LRT | Df | p-val |
| Without random eff. | 12 | -24987.4 | 49998.8 | - | - | - |
| (1 + Limb \| infant) | 10 | -25335.61 | 50691.22 | 696.43 | 2 | <.001 |

#####

##### **Table S3. Comparison of model structures and fit indices for the main analysis.** Model M8 (grey shadow) was chosen for analysis. Abbreviations: N (numbers of parameters in model), AIC (Akaike's Information Criterion), BIC (Bayesian Information Criterion), logLik (logarithm of the likelihood), Dev (Deviance, lack of fit), Df (Degrees of Freedom), and 𝜒^2^ (Chi-square statistic). The better model of the two tested is highlighted in grey.

|  | **Fixed Effects** | **Random Effects** | **Test** | **N** | **AIC** | **BIC** | **logLik** | **Dev** | **𝜒2** | **Df** | **p_val** |
| --- | --- | --- | --- | --- | --- | --- | --- | --- | --- | --- | --- |
| M1 | Time Point | (1 \| infant) | M1 vs **M2** | 6 | 171955.21 | 172008.25 | -85971.61 | 171943.21 |  |  |  |
| **M2** | **Time Point** | **(1 + Limb \| infant)** |  | **8** | **168329.11** | **168399.83** | **-84156.56** | **168313.11** | **3630.10** | **2** | **< .001** |
|  |  |  |  |  |  |  |  |  |  |  |  |
| M2 | Time Point | (1 + Limb \| infant) | M2 vs **M3** |  |  |  |  |  |  |  |  |
| **M3** | **Time Point + Time Window** | **(1 + Limb \| infant)** |  | **10** | **167786.55** | **167874.95** | **-83883.28** | **167766.55** | **546.56** | **2** | **< .001** |
|  |  |  |  |  |  |  |  |  |  |  |  |
| M3 | Time Point + Time Window | (1 + Limb \| infant) | M3 vs **M4** |  |  |  |  |  |  |  |  |
| **M4** | **Time Point + Time Window+ Limb** | **(1 + Limb \| infant)** |  | **11** | **167783.26** | **167880.50** | **-83880.63** | **167761.26** | **5.29** | **1** | **.021** |
|  |  |  |  |  |  |  |  |  |  |  |  |
| M4 | Time Point + Time Window+ Limb | (1 + Limb \| infant) | M4 vs **M5** |  |  |  |  |  |  |  |  |
| **M5** | **Time Point + Time Window + Limb + Time Point:Time Window** | **(1 + Limb \| infant)** |  | **14** | **167753.22** | **167876.98** | **-83862.61** | **167725.22** | **36.04** | **3** | **< .001** |
|  |  |  |  |  |  |  |  |  |  |  |  |
| **M5** | **Time Point + Time Window + Limb + Time Point:Time Window** | **(1 + Limb \| infant)** | **M5** vs M6 |  |  |  |  |  |  |  |  |
| M6 | Time Point + Time Window+ Limb + Time Point:Time Window +  Time Window:Limb | (1 + Limb \| infant) |  | 16 | 167751.92 | 167893.35 | -83859.96 | 167719.92 | 5.30 | 2 | .071 |
|  |  |  |  |  |  |  |  |  |  |  |  |
| **M5** | **Time Point + Time Window + Limb + Time Point:Time Window** | **(1 + Limb \| infant)** | **M5** vs M7 |  |  |  |  |  |  |  |  |
| M7 | Time Point + Time Window+ Limb + Time Point:Time Window +  Time Point:Limb | (1 + Limb \| infant) |  | 20 | 167754.98 | 167931.77 | -83857.49 | 167714.98 | 10.24 | 6 | .115 |
|  |  |  |  |  |  |  |  |  |  |  |  |
| M5 | Time Point + Time Window + Limb + Time Point:Time Window | (1 + Limb \| infant) | M5 vs **M8** |  |  |  |  |  |  |  |  |
| **M8** | **Time Point + Time Window+ Limb +**  **Time Point:Limb** + **Time Point:Time Window + Limb:Time Window + Time Point:Time Window:Limb** | **(1 + Limb \| infant)** |  | **28** | **167742.38** | **167989.89** | **-83843.19** | **167686.38** | **38.84** | **14** | **< .001** |
